# Repair of Torn Avascular Meniscal Cartilage Using Undifferentiated Autologous Mesenchymal Stem Cells: From In Vitro Optimization to a First‐in‐Human Study

**DOI:** 10.1002/sctm.16-0199

**Published:** 2016-12-15

**Authors:** Michael R. Whitehouse, Nicholas R. Howells, Michael C. Parry, Eric Austin, Wael Kafienah, Kyla Brady, Allen E. Goodship, Jonathan D. Eldridge, Ashley W. Blom, Anthony P. Hollander

**Affiliations:** ^1^ Musculoskeletal Research Unit, School of Clinical Sciences; ^2^ School of Cellular and Molecular Medicine, University of BristolBristolUnited Kingdom; ^3^ Avon Orthopaedic CentreSouthmead Hospital, North Bristol NHS TrustBristolUnited Kingdom; ^4^ Orthopaedic Oncology UnitRoyal Orthopaedic HospitalBirminghamUnited Kingdom; ^5^ CMT LaboratoryNHS Blood and TransplantSpekeLiverpoolUnited Kingdom; ^6^ Institute of Integrative Biology, University of LiverpoolLiverpoolUnited Kingdom; ^7^ Institute of Orthopaedics, University College LondonUnited Kingdom; ^8^ Department of OrthopaedicsBristol Royal Infirmary, University Hospitals BristolBristolUnited Kingdom; ^9^ Azellon LtdLondonUnited Kingdom

**Keywords:** Mesenchymal stem cells, Meniscal cartilage, Tissue engineering, Cell therapy

## Abstract

Meniscal cartilage tears are common and predispose to osteoarthritis (OA). Most occur in the avascular portion of the meniscus where current repair techniques usually fail. We described previously the use of undifferentiated autologous mesenchymal stem cells (MSCs) seeded onto a collagen scaffold (MSC/collagen‐scaffold) to integrate meniscal tissues in vitro. Our objective was to translate this method into a cell therapy for patients with torn meniscus, with the long‐term goal of delaying or preventing the onset of OA. After in vitro optimization, we tested an ovine‐MSC/collagen‐scaffold in a sheep meniscal cartilage tear model with promising results after 13 weeks, although repair was not sustained over 6 months. We then conducted a single center, prospective, open‐label first‐in‐human safety study of patients with an avascular meniscal tear. Autologous MSCs were isolated from an iliac crest bone marrow biopsy, expanded and seeded into the collagen scaffold. The resulting human‐MSC/collagen‐scaffold implant was placed into the meniscal tear prior to repair with vertical mattress sutures and the patients were followed for 2 years. Five patients were treated and there was significant clinical improvement on repeated measures analysis. Three were asymptomatic at 24 months with no magnetic resonance imaging evidence of recurrent tear and clinical improvement in knee function scores. Two required subsequent meniscectomy due to retear or nonhealing of the meniscal tear at approximately 15 months after implantation. No other adverse events occurred. We conclude that undifferentiated MSCs could provide a safe way to augment avascular meniscal repair in some patients. Registration: EU Clinical Trials Register, 2010‐024162‐22. Stem Cells Translational Medicine *2017;6:1237–1248*

**Significance Statement**

Each knee has two menisci (medial and lateral) that sit in between the articular cartilage surfaces found at the ends of the upper‐leg and lower‐leg bones. Tears to the meniscus are a common injury caused by excessive twisting force to the knee. Current standard of care for avascular meniscal tears requires surgical removal of the damaged tissue (meniscectomy). However, meniscectomy compromises knee function and leads to associated clinical consequences such as knee replacement surgery and a negative impact on quality of life. The authors have developed a therapy combining undifferentiated mesenchymal stem cells with a collagen scaffold to drive healing of the meniscal tear, so avoiding meniscectomy. The preliminary clinical data are encouraging and suggest for the first time that repair of avascular meniscal tears is possible.

## Introduction

The menisci are fibrocartilagenous structures in the knee. They have a role in load distribution, stability, lubrication, proprioception, and nutrition of articular cartilage 1, 2, 3, 4, 5. Damage to meniscal cartilage is one of the most common knee injuries 6 and loss of meniscal tissue predisposes to osteoarthritis (OA) 7. Only the outer portion of the meniscus retains a blood supply in the adult and is therefore capable of healing following a tear. Vascularity of the meniscus extends in between 10% and 25% from the periphery 8, 9. The majority of meniscal tears occur in the avascular inner zone and thus do not heal 10. Therefore, the standard treatment of symptomatic meniscal tears is to remove the damaged portion of the tissue (partial meniscectomy). A method of enhancing the healing potential of tears in the avascular portion of the meniscus would decrease the need for partial meniscectomy and could in the long run lead to a reduction in the incidence of OA.

In our previous in vitro studies, we developed the concept of using cells seeded onto an open matrix to drive the integration of soft tissues such as cartilage 11. We went on to show that undifferentiated mesenchymal stem cells (MSCs) seeded onto a collagen scaffold induced integration of two pieces of meniscal tissue and improved tensile strength of the repair compared to controls 12. Differentiation of the MSCs to chondrocytes reduced the potential of the cells to integrate with meniscal tissue. Furthermore successful meniscal healing required interaction between the implanted cells and the resident meniscal chondrocytes 12, suggesting that the MSCs were acting through their trophic capacity 13, 14, 15, 16, 17, rather than through direct synthesis of new tissue.

In the current study, we have optimized the MSC/collagen scaffold and developed it into a cell therapy suitable for clinical evaluation. We describe progress through initial testing in an ovine model to a first‐in‐human study to assess the feasibility, safety and clinical outcome of treatment of patients with avascular meniscal tear.

## Materials and Methods

### Isolation and Expansion of Human Marrow‐Derived MSCs for In Vitro Studies

Bone marrow plugs were collected from the femoral heads of patients undergoing total hip replacement. All patients gave their informed consent and the study was carried out according to local ethical guidelines. Cells were suspended in stem cell expansion medium consisting of low glucose Dulbecco's Modified Eagles Medium supplemented with 10% (v/v) Foetal Bovine Serum (FBS, Thermo Scientific Hyclone, Loughborough, UK, www.fishersci.co.uk), 1% (v/v) Glutamax (Sigma), and 1% (v/v) P/S (Sigma, Poole, UK, www.sigmaaldrich.com). The serum batch was selected to promote the growth and differentiation of MSCs 18. The medium was supplemented also with 10 ng/ml FGF‐2 (Peprotech, London, UK, www.peprotech.com). This growth factor has been previously shown to enhance the MSC proliferation rate in vitro 19, 20, to retain MSCs as undifferentiated cells during proliferation 21, 22 and to enhance chondrogenic differentiation when the FGF‐2 expanded MSCs are subsequently exposed to differentiation conditions 19, 20. The cell suspension was separated from any bone in the sample by repeated washing with media. The cells were centrifuged at 500 g for 5 minutes and the supernatant/fat removed. The resulting cell pellet was resuspended in medium, and then plated at a seeding density of between 1.5 and 2.0 × 10^5^ nucleated cells per cm^2^. These flasks were incubated at 37°C in a humidified atmosphere of 5% CO_2_ and 95% air. Four days were allowed before the first medium change and then the medium was changed every other day until adherent cells reached 90% confluence and were ready for passaging. MSCs were characterized for multilineage differentiation and expression of MSC markers CD105, CD90, and VCAM‐1a and for lack for expression of CD34, as previously described 22.

### Preparation of Sheep Meniscal Cartilage

Natural Fibrocartilage cylinders (5.0 mm in diameter and 3.0 mm thick) were harvested from the avascular (white zone) of ovine menisci using a dermal biopsy punch. They were rinsed and incubated with phosphate buffered saline (Invitrogen Ltd, Paisley, UK, www.thermofisher.com) containing 10% (v/v) Penicillin G (10.000 units per ml)/Streptomycin (10.000 μg/ml) antibiotic mixture (P/S; Sigma) and 1% (v/v) Amphotericin B (250 µg/ml; Sigma) for 20 minutes. Viability of the fibrocartilage disks was maintained by culture in basic medium containing Dulbecco's modified Eagle's medium (DMEM, Sigma) with 10 mM Hepes buffer (Sigma), P/S, 1% (v/v) nonessential amino acids (NEAA; Sigma), 1% (v/v) Glutamax (Sigma), and 10% amphotericin B (Sigma) at 37°C in a 5% CO_2_ environment. The explants were used in the integration experiments within 3 days of culture.

### Cell Seeding

Collagen Scaffolds (Ultrafoam Collagen Sponge; Bard, UK, www.barduk.com) were cut into 6‐mm diameter discs and seeded with human MSCs at a concentration of 1 × 10^6^ cells per cm^2^. The suspension was loaded drop wise onto the scaffold placed in ultralow attachment wells of a 24‐well plate (Corning, Acton, USA). After 4 hours, 1.5 ml of expansion medium containing 10 ng/ml FGF‐2 (Peprotech) was added and changed daily. Seeded scaffolds were incubated for 48 hours at 37°C in an orbital shaker at 50 rpm.

### Differentiation Potential of Sheep MSCs

Sheep MSCs were grown in monolayer until 50%–70% confluent prior to osteogenic differentiation or 100% confluent prior to adipogenic differentiation. In both cases, control cells were then cultured in α‐MEM (Invitrogen) basal medium containing 10% FBS, 100 units per ml penicillin, 100 µg/ml streptomycin (all from Sigma), and 2 mM Glutamax‐I (Invitrogen). Cells stimulated to undergo differentiation were cultured in basal medium containing either Osteogenic Supplement or Adipogenic Supplement (R&D Systems, Abingdon, UK, www.rndsystems.com) for 21 days. Following osteogenic differentiation cells were fixed in 70% ethanol and stained with 40 mM alizarin red S (Sigma), pH4.1, for 5 minutes. Following adipogenic differentiation cells were fixed in 4% paraformaldehyde and stained with 0.3% oil red O (Sigma) for 30 minutes.

The chondrogenic capacity of sheep MSCs were assessed by performing three‐dimensional cartilage tissue engineering, as previously described 18, 22, 23. Briefly, 300,000 cells were loaded drop‐wise onto 5 mm diameter × 2 mm thick polyglycolic acid scaffold discs (Biomedical Structures) which had been precoated with 100 μg/ml fibronectin (Sigma). Constructs were then cultured in chondrogenic differentiation medium consisting of DMEM, containing 4,500 mg/l glucose (Sigma), supplemented with 10 ng/ml transforming growth factor‐β3 (TGF‐β3; R&D Systems), 100 nM dexamethasone, 80 µM ascorbic acid 2‐phosphate, 1 mM sodium pyruvate, 100 units per ml penicillin, 100 µg/ml streptomycin (all from Sigma), 1% insulin‐transferrin‐selenium‐G (ITS), and 2 mM Glutamax‐I (both from Invitrogen). After 7 days, the medium was further supplemented with 10 µg/ml bovine pancreatic insulin (Sigma) until the end of culture. The constructs were incubated at 37°C for a total of 35 days on a rotating platform and medium was changed every 3 days.

### Assembling and Culture of Constructs

**Figure 1:**
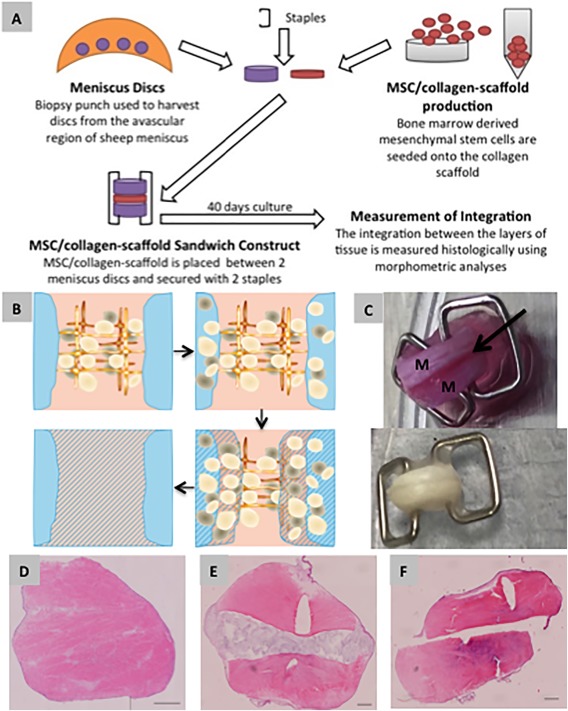
Potency testing of the mesenchymal stem cells (MSCs)/collagen‐scaffold in vitro. **(A)**: Diagram of method for the in vitro potency assay. Human MSCs are seeded onto collagen scaffold to create the MSC/collagen‐scaffold which is then implanted between two discs of sheep meniscal tissue. The three layers are clipped together using staples and then cultured for 40 days. **(B)**: The presumed mode of action of the MSC/collagen‐scaffold, based on previous studies. Undifferentiated MSCs migrate out of the collagen membrane into the surrounding meniscal tissue where their trophic interaction with endogenous meniscal cells leads to a remodeling across the interface between the meniscal surfaces. **(C)**: Macroscopic image of the MSC/collagen‐scaffold potency assay system at the start (upper photograph) end (lower photograph, postfixation for histology) of 40 days of culture. M = meniscus. Arrow shows position of MSC/collagen‐scaffold. **(D)**: Example of successful integration of meniscal tissue in vitro with no histological evidence of a demarcating border. **(E)** Example of apposition of the meniscal tissue in vitro with evidence for alignment of the implant with meniscal tissue but no loss of the demarcating border. **(F)**: Example of disintegration of the meniscal tissue in vitro with a complete lack of interaction between the two pieces of meniscus. For (D‐F) the tissue is stained with haematoxylin and eosin. Scale bar indicates 500 µM. Abbreviation: MSC, mesenchymal stem cell.

Sandwich constructs of two ovine fibrocartilage discs interposed with a seeded scaffold were assembled using skin clips (Fig. 1A, 1C) and cultured in vitro in ultralow attachment 6‐well plates (Corning, Acton, USA) in expansion medium with 10 ng/ml FGF‐2 (Peprotech) for 7 days followed by culture in an integration medium consisting of high glucose DMEM containing 10% (v/v) FBS (Thermo Scientific Hyclone), 1% (v/v), Glutamax, 1% (v/v) P/S, insulin (10 μg/ml; Sigma), and ascorbate‐6‐phosphate (50 μg/ml; Sigma) for 33 days. The medium was replenished twice every week. The constructs were incubated at 37°C on a rotating platform throughout the culture period. At the end of culture, the constructs were prepared for histological analysis by fixation in 10% (v/v) neutral buffered formalin.

### Histological Analysis and Histomorphometry

Constructs fixed in 10% (v/v) neutral buffered formalin were dehydrated and paraffin embedded. Samples were cut into 4‐µm sections and stained with hematoxylin and eosin (H&E) for the study of morphological details. All histological sections were photographed using a digital spot camera (Diagnostics Instruments, Sterling Heights, MI, USA, www.spotimaging.com) and histomorphometric analysis was performed using ImagePro Discovery software (Media Cybernetics, Wokingham, UK). Two perpendicular sections, one at the edge and another one at the center of each construct were used for histomorphometric analysis. The entire length of the implant/meniscus interface was measured with a cursor using a computer mouse to assess the integration. The % integration repair index was determined as we have previously described 11, 12.

### Preclinical Studies in Sheep

Thirty skeletally mature (>2 years old) female sheep were purchased from an approved supplier and were assigned randomly to one of three treatment groups: ovine‐MSC/collagen‐scaffold, scaffold only (no cells) or suture only (no implant). There were five animals in each group to provide appropriate statistical power and the experiment included two replicates of each group, one set with a 13‐week end point and the other set with a 6‐month end point. Details of operative procedures for implantation of the ovine‐MSC/collagen‐scaffold are described in the Supporting Information.

### Tumorogenicity

The tumorogenicty of human MSCs was assessed using the anchorage‐independent growth assay 24, 25. When passage 0 and passage 2 MSCs had reached 80% confluence cells they were trypsinised, counted and diluted to make a cell suspension containing 1.5 × 10^5^ cells per ml of growth media appropriate to the cell. 2.5 ml of cell suspension was added to 5‐ml seeding layer and 1.5‐ml was pipetted onto each petri dish containing the soft agar base layer. This gave a final seeding density of 5 × 10^4^ cells per ml. Five dishes were set up per cell line. The seeded petri dishes were chilled for 20 minutes at 2°C–8°C and then transferred to a humidified incubator at 37°C, 5% CO2. Cells in agar were fed at day 7 of the 14‐day tumorigenicity test. On each occasion a tumorigenicity test was set up for MSC aspirate cells, a corresponding tumorigenicity test was established for HeLa (positive control) and WI‐38 (negative control) cells.

### First‐in‐Human Trial of the Autologous MSC/Collagen‐Scaffold Implant

Details of the manufacture of the human autologous MSC/collagen‐scaffold implant are described in the Supporting Information. Implantations in five patients were performed over an 8‐month period. All the patients were followed up for 24 months or until failure of the implant, resulting in meniscectomy. Four patients were male, the median age at surgery was 37 years (range of 30–38). The median body mass index (BMI) was 25 kg/m^2^ (interquartile range [IQR] 25–26). The mechanism of injury was low energy in all cases with four occurring during activities of daily living and one occurring during sporting activity. All patients had isolated medial meniscal tears in the avascular zone with intact anterior cruciate ligaments. The median passive flexion range of movement was 130° (130°–130°) in the affected knee. This compared to a median passive flexion of 140° (130°–140°) in the contralateral knee. Four patients were employed in manual jobs and one in a desk based job. The median duration of symptoms prior to recruitment was 4 months (IQR, 2‐6). Two patients were current smokers with one an ex‐smoker. According to the International Knee Documentation Committee (IKDC) descriptions, all meniscal tears were complete; four were located in zone 2 of the meniscus and one in zone 3. Three of the tears involved the posterior, mid, and anterior third of the meniscus and two posterior and mid third only. Three were bucket handle tears, one was a bucket handle tear with a radial extension to the inner border of the meniscus and one was a vertical flap tear. All were nondegenerative tears with a median length of 30 mm (IQR, 20–40).

Bone marrow was collected from patients as described in the section below. Upon receipt at the manufacturing site each bone marrow aspirate was mixed with 20 ml of freshly prepared Complete Medium consisting of Dulbecco's Modified Eagle's Medium—low glucose (DMEM LG; Sigma) containing 10% FBS (Invitrogen), 4.5% Glutamax (Invitrogen) and 5 ng/ml FGF‐2 (Peprotech). The resulting marrow‐medium mix was distributed between five 175‐ml tissue culture flasks containing 24 ml of complete media by the addition of 4 ml of mix per flask. Cultures were incubated undisturbed for 4 days at 37°C in 5% CO_2_ and 95% air. After which time, the cells were maintained by media exchange on days 4, 7, and 10. After 13 days, the unpassaged cells were harvested using 0.25% trypsin‐EDTA (Invitrogen) and their identity and purity confirmed by immunohistochemical analyses of positive and negative markers. Positive identity as MSCs required >80% expression of both CD105 and CD90. Confirmation of purity required <10% expression of CD34 and CD45, indicating minimal contamination with haematopoietic stem cells. The cells were then seeded onto a collagen matrix (Avitene Ultrafoam collagen sponge, an absorbable hemostat derived from bovine corium and consisting mainly of Type I collagen) at a dose of 10^6^ cells per cm^2^ of scaffold material, and incubated at 37°C in 5% CO_2_/air for approximately 6 hours (range, 5–7.5 hours) after which the sponge was immersed in hypothermosol (BioLife Solutions, Bothell, WA, USA). The dose had been identified as optimal by Pabbruwe et al. 11, 12.

## Results

### Standardization and Optimization of MSC/Collagen‐Scaffold Technology In Vitro

Building on our previous work 11, 12, we established a standardised in vitro potency assay for integration of meniscal cartilage that we used for all optimization and validation studies (Fig. 1). Using the potency assay we seeded human MSCs into the collagen scaffold at a range of cell doses and identified the optimal dose in the assay as 300 × 10^3^ per scaffold, equivalent to a density of 1 × 10^6^ MSCs per cm^2^ (Supporting Information Fig. S1A). Although there was a significant dose‐response relationship (one‐way ANOVA) there was a lower efficacy at higher seeding densities with no significant integration at the highest two doses compared with the unseeded control. The reason for this not known but could be related to reduced cell motility hen MSCs are in contact with each other at high density, so limiting their capacity to migrate into the surrounding tissue. This cell migration is an important aspect of the mode of action of the construct as we have previously described 11, 12. Therefore the seeding density of 1 × 10^6^ MSCs per cm^2^ was adopted for all subsequent in vitro experiments as well as for the preclinical and clinical studies. In order to establish the minimum incubation time required for MSCs to attach to the collagen scaffold, we made MSC/collagen‐scaffold constructs and measured the number of attached and unattached cells at various time points after initial seeding and in this way we estimated that a minimum of 5 hours of incubation at 37°C is needed to achieve complete attachment of the MSCs to the scaffold (Supporting Information Fig. S1B).

### Validation of Sheep MSCs for use in Preclinical Testing

To avoid species‐species effects that could confound the outcomes if we were to use human MSCs in ovine preclinical testing and to model the autologous nature of the therapeutic more accurately, a homologous ovine construct was produced for use in the sheep studies. Bone marrow stromal cells from ovine hip aspirates were successfully isolated using adherence to tissue culture plastic and once after harvesting they were found to have the capacity for adipogenic and osteogenic differentiation (Supporting Information Fig. S2A–S2D) as well as to create three‐dimensional cartilage in a standard chondrogenic tissue engineering protocol (Supporting Information Fig. S2E). Using the standard potency assay for meniscal cartilage integration we tested ovine bone marrow derived MSCs at the optimal cell density of 1 × 10^6^ cells per cm^2^ and found the results (Supporting Information Fig. S3) to be comparable with our previous studies using human MSCs 12.

### Preclinical Efficacy Studies

**Figure 2:**
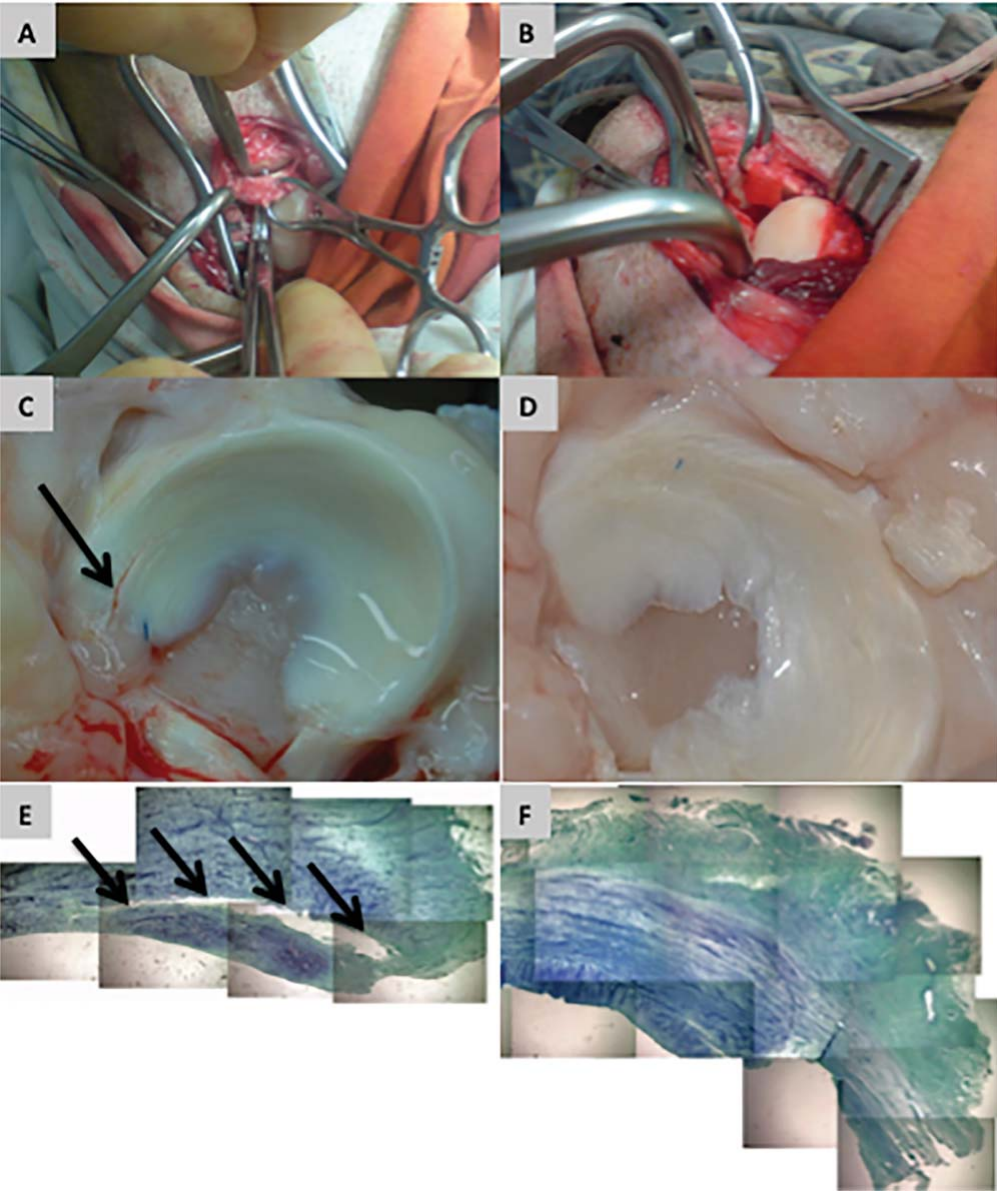
Sheep model for preclinical testing of the efficacy and safety of an ovine‐mesenchymal stem cells (MSCs)/collagen‐scaffold. **(A)**: A full‐depth lesion is created in the avascular zone of the anterior horn of the meniscus in a sheep stifle joint. **(B)**: An ovine‐MSC/collagen‐scaffold made using autologous MSCs is implanted into the fresh lesion and then sutured into position. **(C)**: Macroscopic appearance of a sheep meniscus 13 weeks after implantation of ovine‐MSC/collagen‐scaffold showing apparent failure of repair of the lesion. **(D)**: Macroscopic appearance of a sheep meniscus 13 weeks after implantation of ovine‐MSC/collagen‐scaffold showing apparently successful repair of the lesion. **(E)**: Example of failure of repair by ovine‐MSC/collagen‐scaffold 13 weeks after implantation. **(F)**: Example of successful repair by ovine‐MSC/collagen‐scaffold 13 weeks after implantation. For (E) and (F) the images are compilations of photographs of histological sections of meniscus stained with toluidine blue (Final magnification ×100).

Groups of *n* = 5 sheep were treated with ovine‐MSC/collagen‐scaffold, collagen scaffold only or suture only (Fig. 2). Three out of five sheep (60%) were successfully healed at 13 weeks compared with no healing in any sheep in either of the control groups (Table 1 and Supporting Information Table S1). The difference in outcome between the groups was significant (*p* < .0235; chi‐squared test). However by 24 weeks, none of these animals in any of the groups were free of lesions (Table 1 and Supporting Information Table S1), suggesting that any early repair was unable to survive long‐term in vivo loading of the stifle joint by the sheep.

**Table 1:** Comparison of ovine‐MSC/collagen scaffold with controls for the treatment of torn avascular meniscus in a sheep model

Treatment group	aNumber/proportion healed at 3 months	aNumber/proportion healed at 6 months
Ovine‐MSC/collagen scaffold	3/5 (60%)*	0/5 (0%)
*Collagen scaffold*	0/5 (0%)	0/5 (0%)
*Suture Only*	0/5 (0%)	0/5 (0%)

Sheep were treated with ovine‐MSC/collagen scaffold, collagen scaffold (cell‐free membrane), or suture only and allowed to recover for either 13 weeks or 6 months. There were five sheep for each treatment group at each time point.

a Results shown as the number and proportion of sheep with no meniscal lesion in each group of five animals. Examples of macroscopic and histological outcome can be seen in Figure 2 and raw data for all sheep can be seen in Supporting Information Table ST1. *p < .0235 compared with other treatment groups at the 13‐week time point (chi‐squared test).

### Preclinical Toxicology Studies

In order to ascertain the safety of the MSC/collagen‐scaffold we measured the irritancy of the implant in different parts of the stifle joint according to British Standard EN ISO 10993‐6:2007. The ovine‐MSC/collagen‐scaffold implant and the cell‐free collagen scaffold both caused moderate irritation to the meniscus at 13 weeks while suturing alone caused slight irritancy (Supporting Information Table S2). There were no signs of irritancy of the meniscus by 24 weeks and there was no irritation of the articular cartilage, synovium or popliteal lymph nodes at any time point (Supporting Information Table S2). As a further assessment of safety, human MSCs were tested in vitro for tumorogenicity using the anchorage‐independent colony formation method. This method determines the capacity of cells to proliferate and form colonies when suspended in agar and prevented from attaching to the plastic surface of the culture vessel. Tumor‐forming cells will grow well under these conditions whereas nontransformed cells require attachment to the plastic surface for colony formation and growth. Cells were tested at the end of passage 0 (equivalent to the intended clinical therapeutic) and at the end of passage 2 (i.e., after further expansion in vitro well beyond the intended therapeutic product). There was no evidence of tumor‐forming potential for human MSCs at either passage 0 or 2 whereas Hela cells (positive control) were consistently tumor‐forming and Wi‐38 cells (negative controls) were not (Supporting Information Table S3).

### Production of Autologous Human‐MSC/Collagen‐Scaffolds for Clinical Use

For the clinical study, Human MSCs were isolated from bone marrow that was seeded into tissue culture flasks and cultured in the presence of FGF‐2 for exactly 13 days, without passage. Under these conditions it was possible to routinely generate an MSC population that was free of contamination by red blood cells (Supporting Information Fig. S4). In preliminary experiments, the harvested passage 0 MSCs were found to be positive for a range of typical MSC cell‐surface markers, including CD105, VCAM‐1a, and CD49a and free from contamination by haematopoetic stem cells as judged by the low expression of CD34 (Supporting Information Fig. S5). As the aim was to implant MSCs that were still undifferentiated, we also monitored the expression of nucleostemin, which we have previously shown to be uniquely expressed by undifferentiated MSCs and downregulated upon differentiation 22. MSCs produced using our protocol showed the typical nucleolar location of nucleostemin (Supporting Information Fig. S5G). When deriving MSCs for implantation in patients we used release criteria that were agreed with the regulatory authorities. This included the immunohistochemical detection of >80% cells positive for two MSC markers, both CD105 and CD90 (Supporting Information Fig. S6A, S6B). We also required immunohistochemical detection of <10% cells positive for two haematopoetic stem cell markers, CD45 and CD34. Immunohistochemical detection could be verified by Flow cytometry for all four of these markers (Supporting Information Fig. S6C, S6D). Table 2 shows the characteristics of MSCs produced from bone marrow from each of the five patients taking part in the clinical trial, confirming that there was a good cell yield, high viability and that the release criteria were achieved in all five cases.

**Table 2:** Preimplantation cell production quality data for each patient

Patient	aConfluency at harvest (%)	Total cell yield (×10^6^)	Cell viability at harvest (%)	Immunohistochemistry scores			
				% CD105+ cells^b^	% CD90+ cells^b^	% CD34+ cells^c^	% CD45+ cells^c^
1	90; 90	29.6	97.8	98	94	3	3
2	55; 70	13.3	98.8	96	94	1	6
3	50; 55	27.3	99.1	98.5	90.5	1	2.5
4	70; 70	10.5	99.5	98	82.5	0	2
5	80; 85	29.9	99.0	99	85.5	0	0.5

MSCs were isolated from bone marrow by culture on tissue culture plastic until the end of passage 0.

a Results shown as two separate readings by different observers.

b minimum of 80% + cells required for product release.

c no more than 10% + cells allowed for product release.

### First‐in‐Human Trial Patient Outcomes

**Figure 3:**
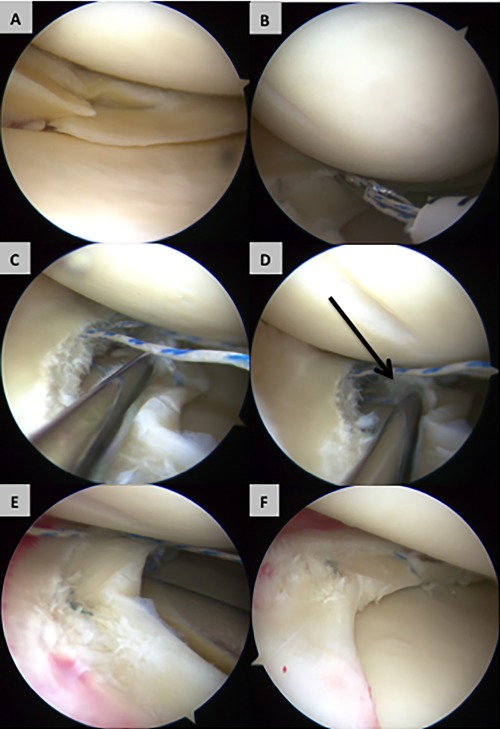
Intraoperative arthroscopic images from one patient showing the method of implantation of human‐mesenchymal stem cells (MSCs)/collagen‐scaffold. **(A)**: Photograph of a bucket handle tear in the white zone (avascular) meniscus with radial extension following reduction of tear. **(B)**: The first stage of treatment is positioning of a vertical mattress suture, loosely across the tear. **(C)**: The human‐MSC/collagen‐scaffold inserted through the arthroscope and then **(D)** inserted into the lesion (arrow). **(E)**: The suture is pulled tight to close the meniscal tissue around the human‐MSC/collagen‐scaffold. **(F)**: Lesion site at the end of the implantation procedure with the human‐MSC/collagen‐scaffold fixed in position in the middle of the sutured tear.

**Figure 4:**
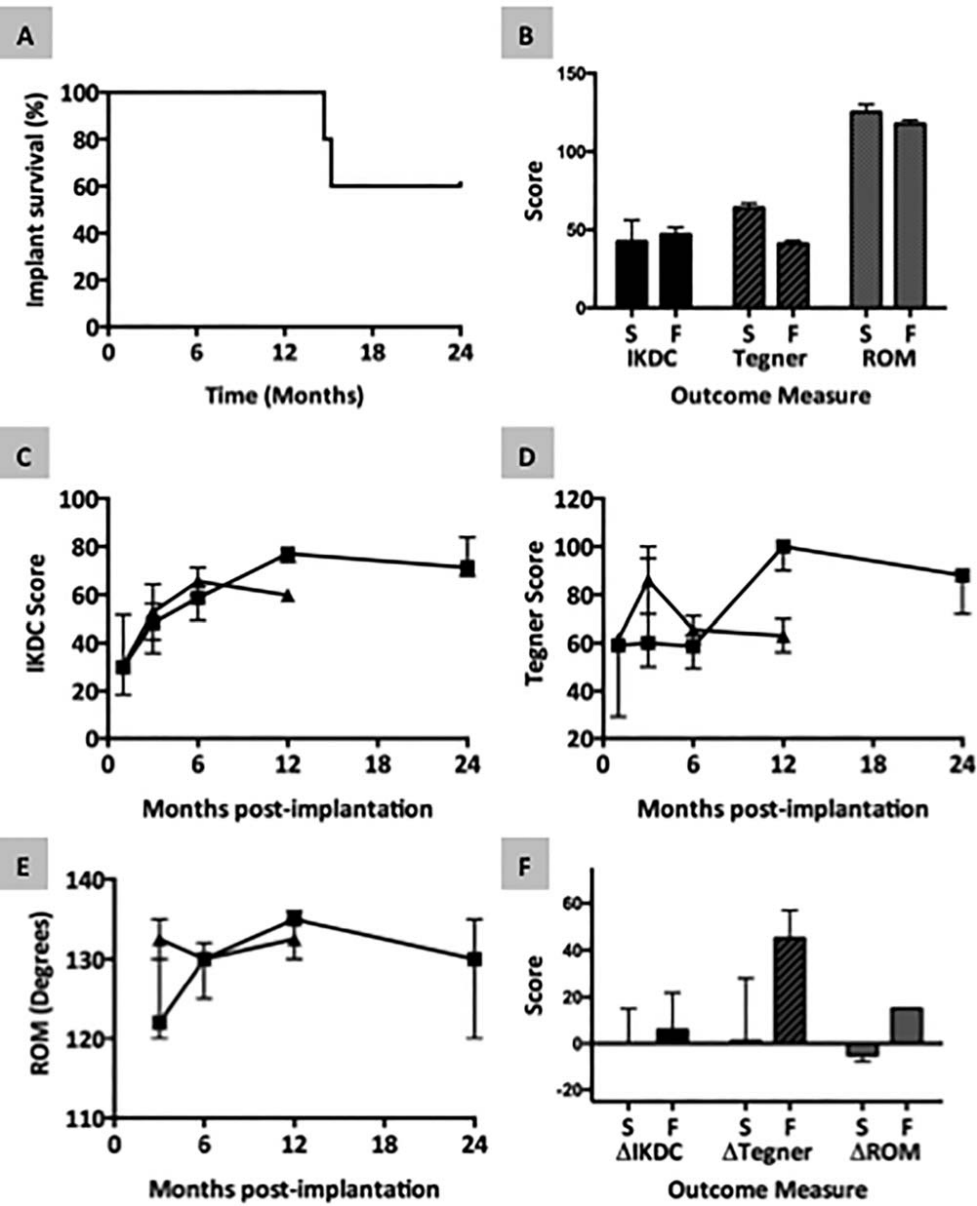
Assessment of the clinical outcome of human‐mesenchymal stem cells (MSCs)/collagen‐scaffold therapy. **(A)**: Survival of the implanted human‐MSC/collagen‐scaffold was determined as length of time before mensicectomy, if needed. **(B)**: Baseline knee function data. The International Knee Documentation Committee (IKDC) score, Tegner‐Lysholm score and range of motion (ROM) were recorded for the affected knee prior to surgery. Data are shown for the three patients in whom the implant survived for 24 months (bars marked S) and the two patients in whom the implant failed and meniscectomy was required (bars marked F). Each bar shows the median and interquartile range (IQR). **(C–E)**: Changes in knee function scores over time postimplantation. The IKDC score (C), Tegner‐Lysholm score (D) and ROM (E) were recorded for the affected knee at multiple time‐points postsurgery. In each graph data are shown separately for the three patients in whom the implant survived for 24 months (squares) and the two patients in whom the implant failed (triangles). Each point shows the median and IQR. **(F)**: Changes in knee function scores from preimplantation to 3 months postimplantation. Data are shown for the three patients in whom the implant survived for 24 months (bars marked S) and the two patients in whom the implant failed (bars marked F). Each bar shows the median and IQR. Abbreviations: IKDC, International Knee Documentation Committee; ROM, range of motion.

Arthroscopy was performed as described Under Materials and Methods and as illustrated in Figure 3. A movie showing the arthroscopic procedure in one of the patients can be seen in Supporting Information (Online Video 1) and additional images of meniscal tears before and after implantation of cell bandage are shown in Supporting Information Figure S7. Of the five patients treated, the implant survived without any further treatment needed in three cases whereas two subsequently developed recurrent symptoms (pain, swelling, and locking in the knee) at around 15 months postimplantation in both cases, leading to treatment with meniscectomy (Fig. 4A). In the first failed case, the appearance at arthroscopy was consistent with a repeat tear at the site of the meniscal repair. In the second failed case, the appearance was consistent with partial healing of the tear in the posterior portion of the tear but with no healing in the anterior portion. In both these cases, the loose meniscal tissue was removed and debrided to a stable rim. No patients reported any serious adverse events or complications other than recurrence of symptoms. In the two patients where the implant eventually failed, the baseline (preimplantation) Tegner‐Lysholm score and range of motion (ROM) of the knee were slightly lower than for the three patients where the implant survived for the entire study period (Fig. 4B). There was no difference in the IKDC score between these groups.

The three patients who were successfully treated showed improvements in all clinical scores over the first 12 months and these changes were maintained between 12 and 24 months (Fig. 4C–4E). In contrast, the two patients in whom the implant failed at 15 months showed no improvement in clinical scores between the 6‐ and 12‐month time points (Fig. 4C–4E). Interestingly, the changes in Tegner‐Lyshom score and ROM between baseline preimplantation measurement and 3 months postimplantation were greater for the two patients where the implant failed than the three patients who were successfully treated (Fig. 4F).

Supporting Information Table S4 shows the clinical scores of the individual patients over time that were used for repeated measures analysis. One patient in whom a meniscectomy was required due to recurrent symptoms at 15 months postoperation declined to provide clinical outcome scores at the 24‐month follow‐up despite multiple invitations to do so and therefore was excluded from the repeated measures analysis. When the repeated measures for the remaining four patients were considered, there was significant clinical improvement as measured by the IKDC score (*p* = .002) and the Tegner Lysholm score (*p* = .005).

**Figure 5:**
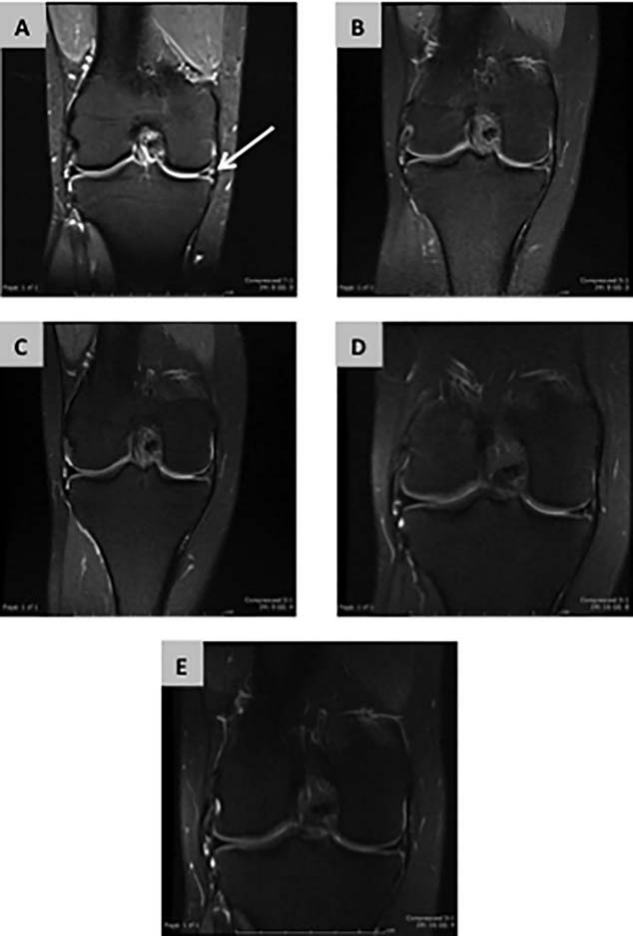
Magnetic Resonance Imaging (MRI) evidence of meniscal repair. Sequential coronal MRI images are show for one of the three patients (patient 3) who were successfully treated using human‐mesenchymal stem cells/collagen‐scaffold therapy. A Preoperative image is shown in **(A)** with an arrow to indicate the torn meniscus. Postoperative images were taken at 3 months **(B)**, 6 months **(C)**, 12 months **(D),** and 24 months **(E)**. MRI data for the other four patients can be seen in Supporting Information Figures S8–S11.

Magnetic Resonance Imaging (MRI) scans show that in the three cases that did not undergo further arthroscopy at 12 months the menisci have not displaced and the abnormally high signal, while persisting, appears to be diminishing with time. An example of the MRI findings over time in one patient is shown in Figure 5 and equivalent results for the other four patients can be seen in Supporting Information (Figs. S8–S11).

## Discussion

Removal of a greater amount of meniscal tissue at meniscectomy is associated with poorer long‐term function 26 and thus attempts to repair meniscal tears in the vascular zone are becoming more common 27, 28. Healing rates of 50% may be achieved when repairing vascularized tears in the presence of concomitant anterior cruciate ligament (ACL) repairs (which favors healing of meniscal repairs) 27, 29. A lower rate of healing is observed when ACL repair is not performed at the same time, when medial rather than lateral meniscal repair is performed and when repairs in the avascular zone of the meniscus are performed 10, 30. Here, we report the first series of isolated medial meniscal tears in the avascular zone treated with MSC therapy. The treatment appears to be safe and has some efficacy with three out of five patients having resolution of symptoms 2 years after surgery. In the three successfully treated patients, sequential postoperative MRI scans show decreased signal intensity over time, which may indicate healing. In all three survivors the MRI confirms that the torn area has not displaced, again suggesting healing.

Therefore the proportion of patients in whom the avascular tear was healed in this small‐scale first‐in‐human study was similar to the proportion of patients successfully treated for tears in the vascularized meniscus using conventional repair methodology 27, 29. The two patients in whom the implant failed had slightly worse baseline clinical data and a more rapid increase in joint function over the first 3 months of the study. In the preclinical sheep model, there was a 60% success rate at 3 months but 100% failure at 6 months. The sheep were free to load their stifle joints from the moment after surgery, unlike the patients, who were trained to gradually increase loading over a period of weeks. Taken together, these observations highlight the potential importance of mechanical factors in the final outcome of treatment and the need to ensure an optimal postoperative rehabilitation regime in any future studies.

Stem cell therapy is in its infancy and we are only gradually understanding how best to use these cells in the clinical setting. While much research effort has concentrated on understanding how we can effectively differentiate MSCs to specific committed lineages 23, 31, 32, 33, 34, 35, 36, 37, 38, 39, 40, 41, there has been growing interest in the trophic effects of the undifferentiated cells mediated through secretion of growth factors 13, 14, 15, 16, 17. Based on our original in vitro observations 12 we hypothesized that combining undifferentiated MSCs with a collagen scaffold, we could deliver the cells directly into the site of injury and maximize the chance of secreted trophic factors driving a tissue repair response. The undifferentiated status of MSCs prepared using our methodology was confirmed by the presence of nucelostemin in a nucleolar location. Nucleostemin was discovered as a nucleolar protein present in both embryonic and adult rat central nervous system stem cells, and several human cancer cell lines. It is abundantly expressed while the cells are proliferating in an early, multipotential state, but it abruptly and almost entirely disappears at the start of differentiation 42. We subsequently showed that it is similarly expressed in undifferentiated MSCs 22. The clinical data reported here provide some support for our original hypothesis and suggest that the predifferentiation of MSCs may not be necessary for tissue repair. The effectiveness of undifferentiated stem cells in healing the avascular meniscus suggests that a similar approach could be used to heal injuries to other soft tissues.

While the work reported here is the first attempt to investigate the short to medium‐term effects of MSC/collagen‐scaffolds in an avascular meniscal cartilage repair setting, our longer‐term goal is to use this technology as a method for preventing the development of OA. It has been understood for many years that meniscectomy substantially increases the risk of developing OA in the operated knee in both animal models 43, 44, 45, 46, 47, 48, 49, 50 and in the human clinical situation 51, 52, 53, 54, 55, 56, 57, 58. Furthermore, the degree of risk of OA increases with increasing amount of meniscus that is removed 56, 58. The menisci play a critical role in mechanical stabilization of the knee and removal of substantial amounts of meniscal tissue is thought to result in abnormal loading of the articular cartilage that would otherwise be protected 58. One study 55 investigated 155 patients for 16 years postmeniscectomy and estimated the risk of developing OA compared with the general population. The relative risk was found to be 7.0 per year for patients with degenerative tears and 2.7 for those with traumatic tears and in this study there was no apparent link to the amount of tissue removed.

Given the above analysis, it seems reasonable to propose that repair of meniscus may allow us to avoid removal of tissue that is likely to prevent, or at least delay, the development of OA. However, until now there has been no technology that could be used to repair the majority of avascular meniscal tears and therefore limited possibility to test the hypothesis. Since repair of red zone (vascular) meniscal tears is now widely used, it may be possible to predict the impact of meniscal repair on the incidence of OA in this patient group. A few studies have indicated the potential for a reduction in OA when the vascular meniscus is repaired rather than removed 59, 60, 61, however, much larger studies will be required to test this hypothesis properly. Therefore, future clinical trials will need to assess both the short‐term repair capability of MSC/collagen‐scaffold and the longer‐term ability of the technique to reduce the risk of developing OA.

Because of the detrimental effects of mensicectomy, a number of experimental approaches have been taken to the treatment of avascular meniscal tears. One paper has provided a meta‐analysis of the outcome of meniscal allograft in 44 clinical trials 62, concluding that “meniscal allograft transplantation is a reliable solution for postmeniscectomy symptoms in selected patients.” Similarly, Warth and Rodkey 63, 64 have recently systematically reviewed 13 studies in which partial meniscectomy was augmented with implantation of cell‐free collagen scaffolds, concluding that the scaffolds provided some improvement compared with mensicectomy alone. However, the common aspect to both these above approaches is that they are used to manage the effects of meniscectomy by replacing the meniscus altogether (allograft) or replacing the removed tissue after partial meniscectomy (collagen scaffolds). The method described here is the first report in humans of an attempt to heal avascular tears as a way of avoiding meniscectomy altogether. It remains to be established if restoring the natural meniscus can prevent or delay the development of OA more effectively than meniscal allograft or postmeniscectomy collagen scaffold implantation.

## Conclusion

Undifferentiated autologous MSCs seeded onto collagen‐scaffolds can be safely implanted into patients with torn avascular meniscus and show some potential for enhancing the regeneration of the damaged meniscus in some patients, so avoiding the need for meniscectomy.

## Author Contributions

M.W.: Conception and design, Provision of study material or patients, Collection and/or assembly of data, Data analysis and interpretation, Manuscript writing, Final approval of manuscript, Other ‐ recruited the patients and undertook the bone marrow harvest; N.H.: Conception and design, Provision of study material or patients, Collection and/or assembly of data, Manuscript writing, Final approval of manuscript, Other ‐ recruited the patients and undertook the bone marrow harvest; M.P.: Provision of study material or patients, Collection and/or assembly of data, Manuscript writing, Final approval of manuscript, Other ‐ recruited the patients and undertook the bone marrow harvest; E.A.: Provision of study material or patients, Collection and/or assembly of data, Manuscript writing, Final approval of manuscript, developed the Good Manufacturing Practice methodology for production of the MSC/collagen‐scaffold; W.K.: Collection and/or assembly of data, Manuscript writing, Final approval of manuscript. Other – designed and optimized the MSC/collagen‐scaffold technology; K.B.: Collection and/or assembly of data, Manuscript writing, Final approval of manuscript; A.G.: Provision of study material or patients, Manuscript writing, Final approval of manuscript; J.E.: Provision of study material or patients, Manuscript writing, Final approval of manuscript, Other ‐ performed all implantation surgery; A.B.: Conception and design, Data analysis and interpretation, Manuscript writing, Final approval of manuscript, Other ‐ had full access to all of the data in the study and takes responsibility for the integrity of the data and the accuracy of the data analysis; A.H.: Conception and design, Financial support, Manuscript writing, Final approval of manuscript, Other ‐ designed, developed and optimised the MSC/collagen‐scaffold technology used in this study, developed the Good Manufacturing Practice methodology for production of the MSC/collagen‐scaffold.

## Disclosure of Potential Conflicts of Interest

A.P.H. is a founder, director of and share‐holder in Azellon Ltd, a University of Bristol spin‐out company. E.A.'s host institution (NHS Blood and Transplant) was a paid contractor for work carried out in the course of the study with funding received from Azellon. M.R.W., N.R.H., M.C.P., J.D.E. and A.W.B.'s host institution (North Bristol NHS Trust) received funding from Azellon for the costs of the clinical study and research nurse support. M.R.W., N.R.H., M.C.P., E.A., J.D.E. and A.W.B. did not directly receive funding or benefits in relation to the study. Neither funding organisation reviewed, commented on or approved the manuscript prior to submission. All authorshad full access to the data. Because of the corresponding author's conflict of interest with respect to Azellon Ltd, A.B. acted as the guarantor, attests that the findings are a true and accurate representation of the study and the final decision to submit was his.

## Supporting information

Supporting Information Fig. S1

Supporting Information Fig. S2

Supporting Information Fig. S3

Supporting Information Fig. S4

Supporting Information Fig. S5

Supporting Information Fig. S6

Supporting Information Fig. S7

Supporting Information Fig. S8

Supporting Information Fig. S9

Supporting Information Fig. S10

Supporting Information Fig. S11

Supporting Information Table S1

Supporting Information Table S2

Supporting Information Table S3

Supporting Information Table S4

Supporting Information Video S1

Supporting Information 1

Supporting Information 2
