# Supplementary figures and images for: Repair of Torn Avascular Meniscal Cartilage Using Undifferentiated Autologous Mesenchymal Stem Cells: From In Vitro Optimization to a First‐in‐Human Study
Source: Stem Cells Transl Med. 2016 Dec 15;6(4):1237–48. doi: 10.1002/sctm.16-0199 (PMC5442845; doi:10.1002/sctm.16-0199)

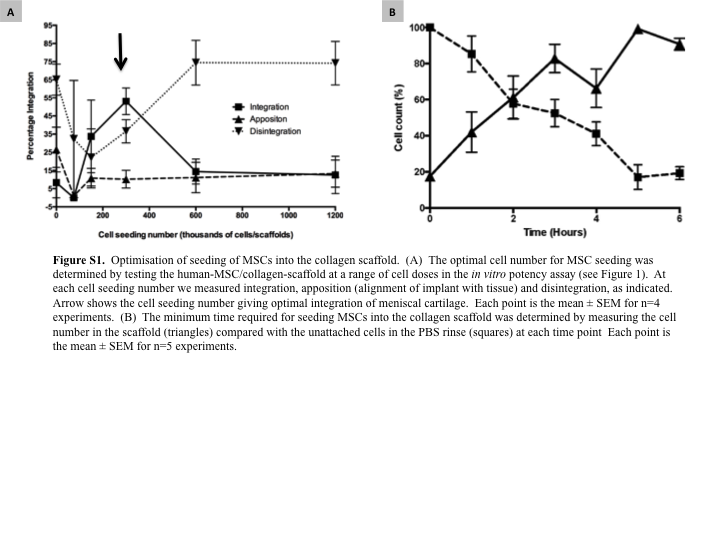

Supplement: Supplementary file 1 — Supporting Information Fig. S1 [file SCT3-6-1237-s001.tiff]

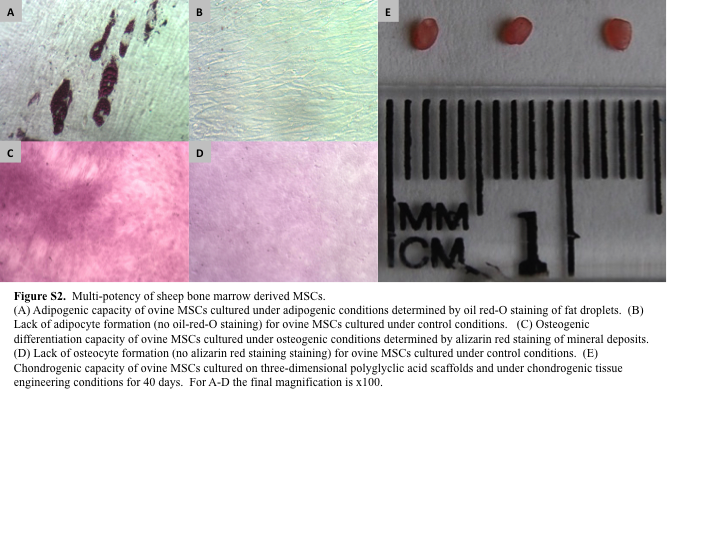

Supplement: Supplementary file 2 — Supporting Information Fig. S2 [file SCT3-6-1237-s002.tiff]

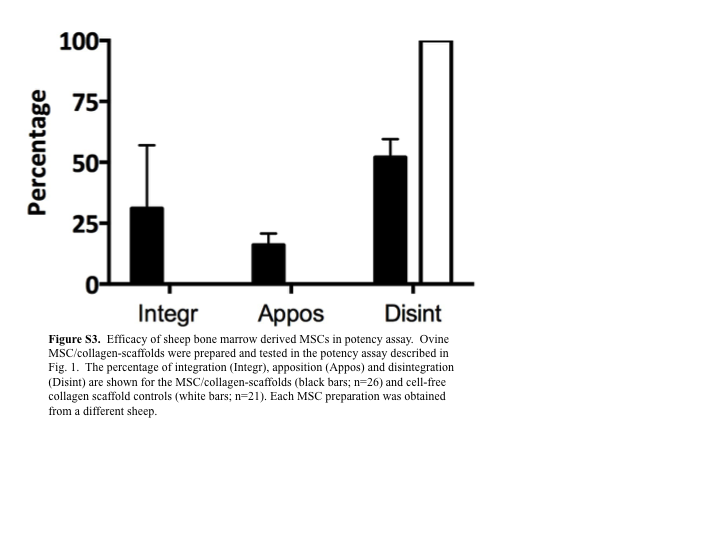

Supplement: Supplementary file 3 — Supporting Information Fig. S3 [file SCT3-6-1237-s003.tiff]

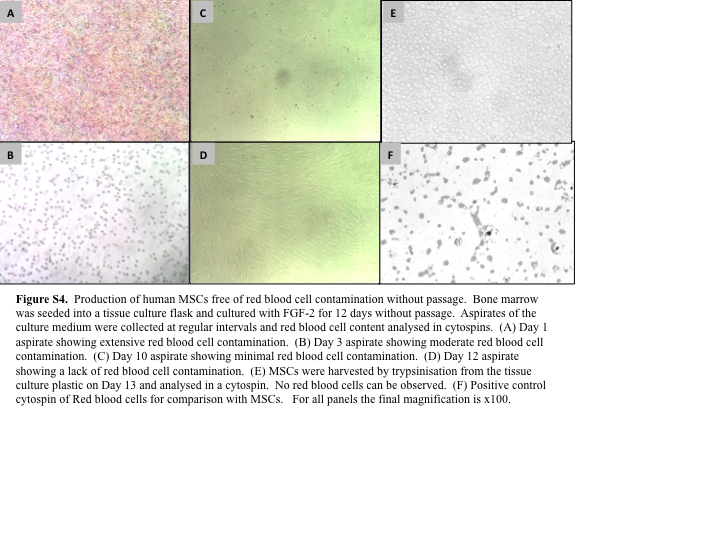

Supplement: Supplementary file 4 — Supporting Information Fig. S4 [file SCT3-6-1237-s004.tiff]

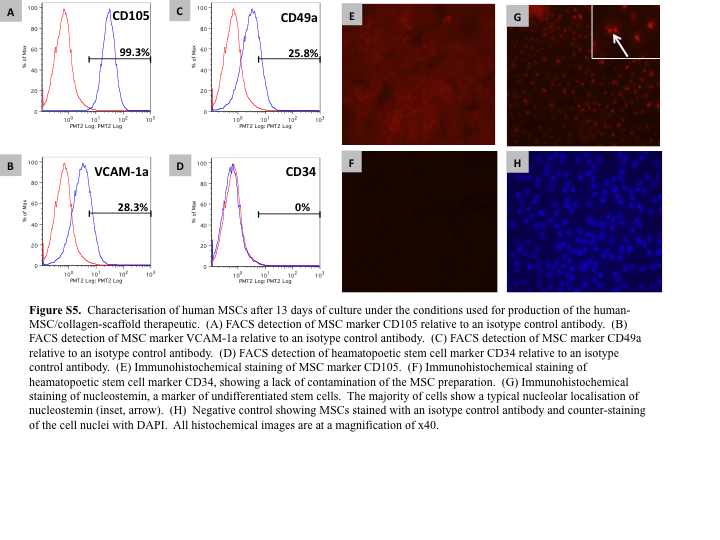

Supplement: Supplementary file 5 — Supporting Information Fig. S5 [file SCT3-6-1237-s005.tiff]

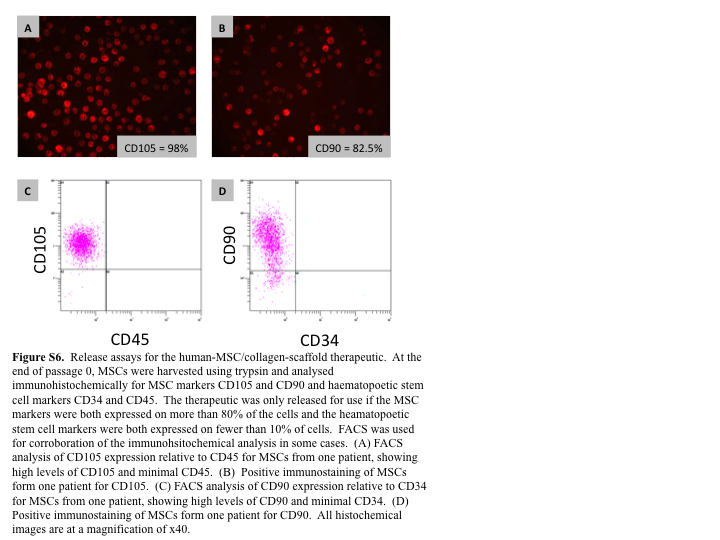

Supplement: Supplementary file 6 — Supporting Information Fig. S6 [file SCT3-6-1237-s006.tiff]

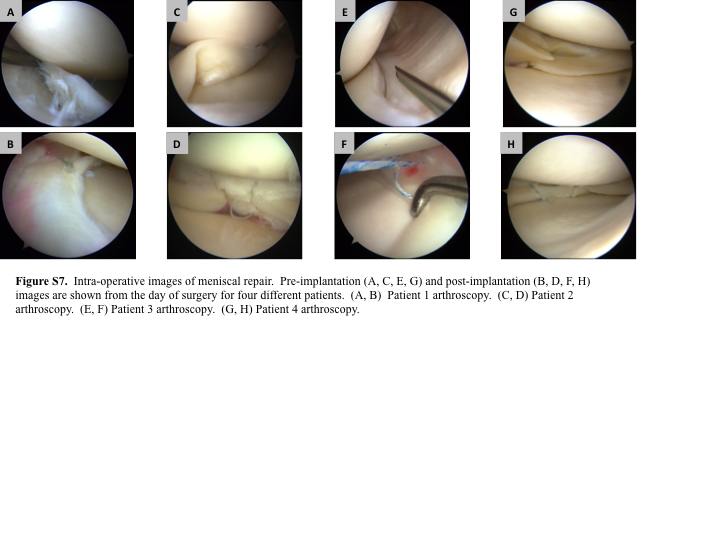

Supplement: Supplementary file 7 — Supporting Information Fig. S7 [file SCT3-6-1237-s007.tiff]

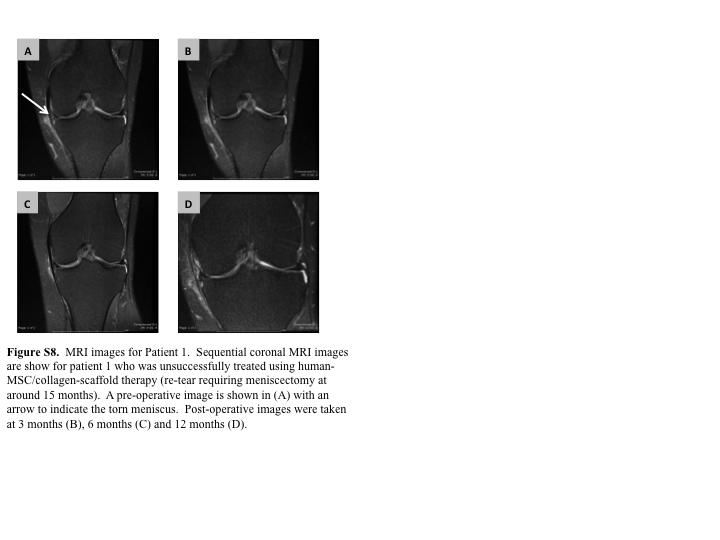

Supplement: Supplementary file 8 — Supporting Information Fig. S8 [file SCT3-6-1237-s008.tiff]

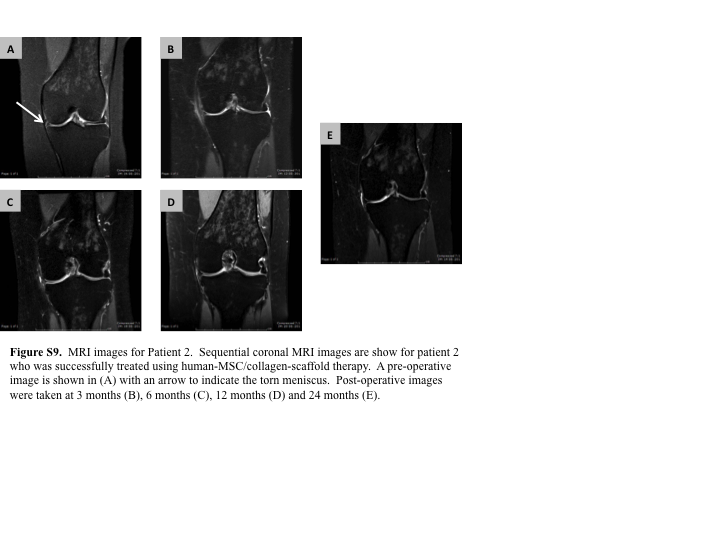

Supplement: Supplementary file 9 — Supporting Information Fig. S9 [file SCT3-6-1237-s009.tiff]

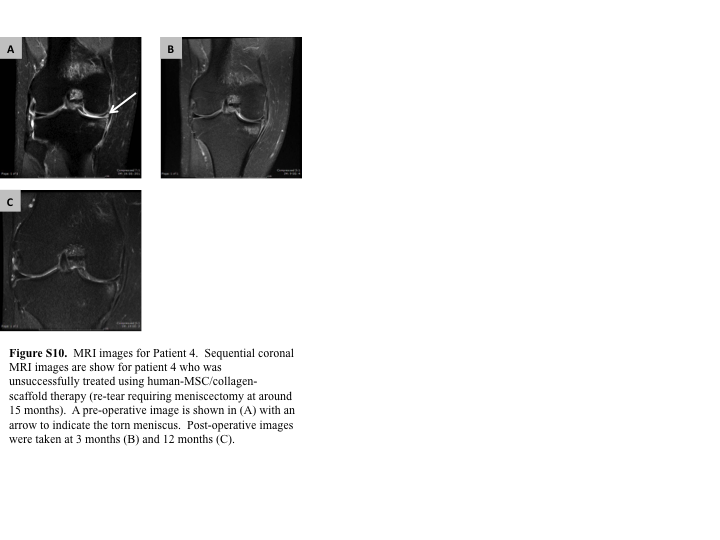

Supplement: Supplementary file 10 — Supporting Information Fig. S10 [file SCT3-6-1237-s010.tiff]

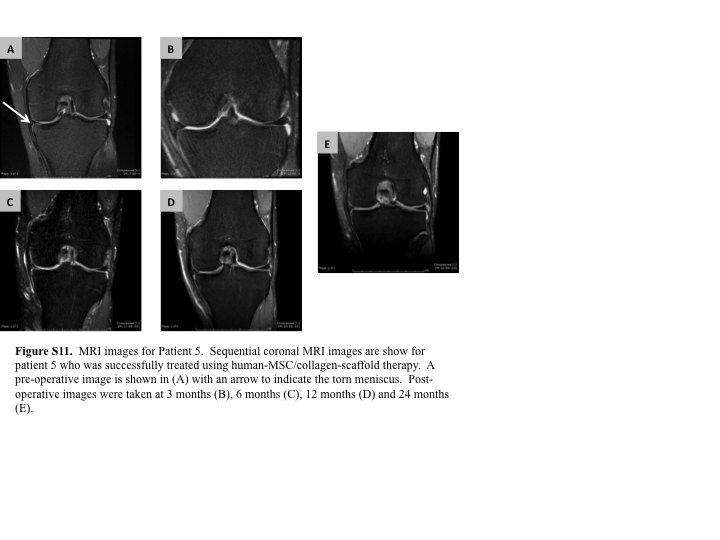

Supplement: Supplementary file 11 — Supporting Information Fig. S11 [file SCT3-6-1237-s011.tiff]
