# Supplementary material for: Repair of Torn Avascular Meniscal Cartilage Using Undifferentiated Autologous Mesenchymal Stem Cells: From In Vitro Optimization to a First‐in‐Human Study
Source: Stem Cells Transl Med. 2016 Dec 15;6(4):1237–48. doi: 10.1002/sctm.16-0199 (PMC5442845; doi:10.1002/sctm.16-0199)
Supplement: Supplementary file 12 — Supporting Information Table S1 [file SCT3-6-1237-s012.docx]

| **Treatment group** | **^a^Lesion size (mm)** | | | | |
| --- | --- | --- | --- | --- | --- |
|  | **^b^Experiment 1** | **^b^Experiment 2** | **^b^Experiment 3** | **^b^Experiment 4** | **^b^Experiment 5** |
| *Suture only*  *13 Weeks* | **6.0** | **3.3** | **3.7** | **5.3** | **9.4** |
| *Suture only*  *6 months* | **9.3** | **9.7** | **9.8** | **8.6** | **5.7** |
| *Cell-free scaffold*  *13 weeks* | **7.4** | **10.2** | **8.0** | **10.9** | **9.4** |
| *Cell-free scaffold*  *6 months* | **10.05** | **11.16** | **9.1** | **5.9** | **8.6** |
| *Cell Bandage*  *13 weeks* | **0** | **9.1** | **0** | **7.5** | **0** |
| *Cell Bandage*  *6 months* | **9.4** | **10.2** | **8.9** | **8.4** | **9.8** |

**Table S1. Raw data for Cell Bandage efficacy study in a sheep model of meniscal repair.** A 10mm lesion was made in the avascular zone of the sheep stifle joint and the lesion was then immediately repaired with a suture alone, implantation of cell-free collagen scaffold or Cell Bandage (collagen scaffold plus cells). Sheep were sacrificed after 13 weeks or 6 month and serial sections of the repair site taken from the upper to lower surface of the meniscus. **^a^**The lesion size was measured microscopically using a morphometric analysis tool at three standardised depths. The results are shown as an average value for each meniscus. **^b^**Each experimental included 6 sheep with one being used for each experimental condition.
