# Supplementary material for: Repair of Torn Avascular Meniscal Cartilage Using Undifferentiated Autologous Mesenchymal Stem Cells: From In Vitro Optimization to a First‐in‐Human Study
Source: Stem Cells Transl Med. 2016 Dec 15;6(4):1237–48. doi: 10.1002/sctm.16-0199 (PMC5442845; doi:10.1002/sctm.16-0199)
Supplement: Supplementary file 13 — Supporting Information Table S2 [file SCT3-6-1237-s013.docx]

| **Treatment group** | **Irritant Score** | | | | | | | |
| --- | --- | --- | --- | --- | --- | --- | --- | --- |
|  | **Meniscus** | | **Articular Cartilage** | | **Synovium** | | **Popliteal Lymph Node** | |
|  | **Mean (SD)** | **Classification** | **Mean (SD)** | **Classification** | **Mean (SD)** | **Classification** | **Mean (SD)** | **Classification** |
| *Suture only*  *13 Weeks* | 7.4  (2.7) | Slight irritant | 0  (0.00) | Non-irritant | 0  (0.00) | Non-irritant | -0.40  (0.89) | Non-irritant |
| *Suture only*  *6 months* | 5.8  (0.45) | Slight irritant | 0.4  (1.26) | Non-irritant | 0  (0.00) | Non-irritant | -0.40  (1.67) | Non-irritant |
| *Cell-free scaffold*  *13 weeks* | 10.6  (5.27) | Moderate irritant | 0  (0.00) | Non-irritant | 0  (0.00) | Non-irritant | -1.60  (1.67) | Non-irritant |
| *Cell-free scaffold*  *6 months* | 7.4  (1.67) | Slight  irritant | 0.4  (1.26) | Non-irritant | 0  (0.00) | Non-irritant | 0.4  (2.61) | Non-irritant |
| *Cell Bandage*  *13 weeks* | 11.8  (5.07) | Moderate irritant | 0.80  (1.69) | Non-irritant | 1.00  (2.83) | Non-irritant | -0.80  (1.79) | Non-irritant |
| *Cell Bandage*  *6 months* | 7.4  (3.71) | Slight  irritant | 0  (0.00) | Non-irritant | 0  (1.41) | Non-irritant | -0.80  (1.79) | Non-irritant |

**Table S2. Evaluation of the irritancy of each treatment group in a sheep model.** Sheep were treated with just a suture, collagen scaffold without cells or Cell Bandage. After either 13 weeks or 6 months the animals were sacrificed and different tissues from the treated stifle joint evaluated histologically for irritancy, according to British Standard EN ISO 10993-6:2007. Results are the mean (SD) for n=5 sheep in each group.
