# Supplementary material for: Repair of Torn Avascular Meniscal Cartilage Using Undifferentiated Autologous Mesenchymal Stem Cells: From In Vitro Optimization to a First‐in‐Human Study
Source: Stem Cells Transl Med. 2016 Dec 15;6(4):1237–48. doi: 10.1002/sctm.16-0199 (PMC5442845; doi:10.1002/sctm.16-0199)
Supplement: Supplementary file 14 — Supporting Information Table S3 [file SCT3-6-1237-s014.docx]

| **MSC sample** | **Treatment Group** | **Evaluation of colony formation and progression** | | | |
| --- | --- | --- | --- | --- | --- |
|  |  | **Passage 0 MSCs** | | **Passage 2 MSCs** | |
|  |  | **Day 7** | **Day 14** | **Day 7** | **Day 14** |
| 1 | MSCs | -ve | -ve | -ve | -ve |
|  | HeLA cells (positive control) | +ve | +ve | +ve | +ve |
|  | WI-38 cells (negative control) | -ve | -ve | -ve | -ve |
| 2 | MSCs | -ve | -ve | -ve | -ve |
|  | HeLA cells (positive control) | +ve | +ve | +ve | +ve |
|  | WI-38 cells (negative control) | -ve | -ve | -ve | -ve |
| 3 | MSCs | -ve | -ve | -ve | -ve |
|  | HeLA cells (positive control) | +ve | +ve | +ve | +ve |
|  | WI-38 cells (negative control) | -ve | -ve | -ve | -ve |
| 4 | MSCs | -ve | -ve | -ve | -ve |
|  | HeLA cells (positive control) | +ve | +ve | +ve | +ve |
|  | WI-38 cells (negative control) | -ve | -ve | -ve | -ve |
| 5 | MSCs | -ve | -ve | -ve | -ve |
|  | HeLA cells (positive control) | +ve | +ve | +ve | +ve |
|  | WI-38 cells (negative control) | -ve | -ve | -ve | -ve |
| 6 | MSCs | -ve | -ve | -ve | -ve |
|  | HeLA cells (positive control) | +ve | +ve | +ve | +ve |
|  | WI-38 cells (negative control) | -ve | -ve | -ve | -ve |
| 7 | MSCs | -ve | -ve | -ve | -ve |
|  | HeLA cells (positive control) | +ve | +ve | +ve | +ve |
|  | WI-38 cells (negative control) | -ve | -ve | -ve | -ve |

**Table S3. Evaluation of the tumorogenicity of the MSC drug substance by anchorage-independent colony formation *in vitro*.** MSCs were isolated from the bone marrow of 7 different patients and cultured under the same conditions used for preparation of Cell Bandage. Cells were harvested at the end of passage 0 and 2 and tested for anchorage-independent colony formation for 7 or 14 days. For each bone marrow sample the MSCs were tested in 5 replicate plates and compared with a single plate for each of the positive and negative controls. Cultures were considered negative when colonies consisted of 1-20 cells and there was progression in less than 2% of cells (CEF <2%).
