# Supplementary material for: Repair of Torn Avascular Meniscal Cartilage Using Undifferentiated Autologous Mesenchymal Stem Cells: From In Vitro Optimization to a First‐in‐Human Study
Source: Stem Cells Transl Med. 2016 Dec 15;6(4):1237–48. doi: 10.1002/sctm.16-0199 (PMC5442845; doi:10.1002/sctm.16-0199)
Supplement: Supplementary file 15 — Supporting Information Table S4 [file SCT3-6-1237-s015.docx]

| **^a^Outcome measure** | | **Ore-operative** | **1 Week** | **1 Month** | **3 Months** | **6 Months** | **12 Months** | **24 Months** | **^b^p value** |
| --- | --- | --- | --- | --- | --- | --- | --- | --- | --- |
| **Active Flexion** | **Median** | **125** | **N/A** | **N/A** | **130** | **130** | **135** | **133** | **0.16** |
|  | **IQR** | **120-125** | **N/A** | **N/A** | **122-130** | **130-130** | **130-135** | **128-136** |  |
| **IKDC**  **Score** | **Median** | **43** | **28** | **30** | **48** | **60** | **74** | **75** | **0.002** |
|  | **IQR** | **43-52** | **26-32** | **28-33** | **41-56** | **59-63** | **60-77** | **70-80** |  |
| **Tegner**  **Score** | **Median** | **49** | **52** | **59** | **72** | **84** | **90** | **88** | **0.05** |
|  | **IQR** | **43-64** | **44-56** | **59-61** | **60-95** | **81-95** | **70-100** | **84-90** |  |

**Table S4. Repeated measures analysis for patients treated with MSC/collagen-scaffold.** Patients were evaluated for active flexion of the treated knee, IKDC score and Tegner-Lysholm score at all the time-points shown. Results are shown for only 4 patients because Patient 1 did not return for the 24 month assessment. **^b^**Friedman test for repeated measure.
