# Supplementary material for: Repair of Torn Avascular Meniscal Cartilage Using Undifferentiated Autologous Mesenchymal Stem Cells: From In Vitro Optimization to a First‐in‐Human Study
Source: Stem Cells Transl Med. 2016 Dec 15;6(4):1237–48. doi: 10.1002/sctm.16-0199 (PMC5442845; doi:10.1002/sctm.16-0199)
Supplement: Supplementary file 17 — Supporting Information 1 [file SCT3-6-1237-s017.docx]

**Supplemental Figure S1.** Optimisation of seeding of MSCs into the collagen scaffold. (A) The optimal cell number for MSC seeding was determined by testing the human-MSC/collagen-scaffold at a range of cell doses in the *in vitro* potency assay (see Figure 1). At each cell seeding number we measured integration, apposition (alignment of implant with tissue) and disintegration, as indicated. Arrow shows the cell seeding number giving optimal integration of meniscal cartilage. Each point is the mean ± SEM for n=4 experiments. (B) The minimum time required for seeding MSCs into the collagen scaffold was determined by measuring the cell number in the scaffold (triangles) compared with the unattached cells in the PBS rinse (squares) at each time point Each point is the mean ± SEM for n=5 experiments.

**Supplemental Figure S2.** Multi-potency of sheep bone marrow derived MSCs.

(A) Adipogenic capacity of ovine MSCs cultured under adipogenic conditions determined by oil red-O staining of fat droplets. (B) Lack of adipocyte formation (no oil-red-O staining) for ovine MSCs cultured under control conditions. (C) Osteogenic differentiation capacity of ovine MSCs cultured under osteogenic conditions determined by alizarin red staining of mineral deposits. (D) Lack of osteocyte formation (no alizarin red staining staining) for ovine MSCs cultured under control conditions. (E) Chondrogenic capacity of ovine MSCs cultured on three-dimensional polyglyclic acid scaffolds and under chondrogenic tissue engineering conditions for 40 days. For A-D the final magnification is x100.

**Supplemental Figure S3.** Efficacy of sheep bone marrow derived MSCs in potency assay. Ovine MSC/collagen-scaffolds were prepared and tested in the potency assay described in Fig. 1. The percentage of integration (Integr), apposition (Appos) and disintegration (Disint) are shown for the MSC/collagen-scaffolds (black bars; n=26) and cell-free collagen scaffold controls (white bars; n=21).

**Supplemental Figure S4.** Production of human MSCs free of red blood cell contamination without passage. Bone marrow was seeded into a tissue culture flask and cultured with FGF-2 for 12 days without passage. Aspirates of the culture medium were collected at regular intervals and red blood cell content analysed in cytospins. (A) Day 1 aspirate showing extensive red blood cell contamination. (B) Day 3 aspirate showing moderate red blood cell contamination. (C) Day 10 aspirate showing minimal red blood cell contamination. (D) Day 12 aspirate showing a lack of red blood cell contamination. (E) MSCs were harvested by trypsinisation from the tissue culture plastic on Day 13 and analysed in a cytospin. No red blood cells can be observed. (F) Positive control cytospin of Red blood cells for comparison with MSCs. For all panels the final magnification is x100.

**Supplemental Figure S5.** Characterisation of human MSCs after 13 days of culture under the conditions used for production of the human-MSC/collagen-scaffold therapeutic. (A) FACS detection of MSC marker CD105 relative to an isotype control antibody. (B) FACS detection of MSC marker VCAM-1a relative to an isotype control antibody. (C) FACS detection of MSC marker CD49a relative to an isotype control antibody. (D) FACS detection of heamatopoetic stem cell marker CD34 relative to an isotype control antibody. (E) Immunohistochemical staining of MSC marker CD105. (F) Immunohistochemical staining of heamatopoetic stem cell marker CD34, showing a lack of contamination of the MSC preparation. (G) Immunohistochemical staining of nucleostemin, a marker of undifferentiated stem cells. The majority of cells show a typical nucleolar localisation of nucleostemin (inset, arrow). (H) Negative control showing MSCs stained with an isotype control antibody and counter-staining of the cell nuclei with DAPI. All histochemical images are at a magnification of x40.

**Supplemental Figure S6.** Release assays for the human-MSC/collagen-scaffold therapeutic. At the end of passage 0, MSCs were harvested using trypsin and analysed immunohistochemically for MSC markers CD105 and CD90 and haematopoetic stem cell markers CD34 and CD45. The therapeutic was only released for use if the MSC markers were both expressed on more than 80% of the cells and the heamatopoetic stem cell markers were both expressed on fewer than 10% of cells. FACS was used for corroboration of the immunohsitochemical analysis in some cases. (A) FACS analysis of CD105 expression relative to CD45 for MSCs from one patient, showing high levels of CD105 and minimal CD45. (B) Positive immunostaining of MSCs form one patient for CD105. (C) FACS analysis of CD90 expression relative to CD34 for MSCs from one patient, showing high levels of CD90 and minimal CD34. (D) Positive immunostaining of MSCs form one patient for CD90. All histochemical images are at a magnification of x40.

**Supplemental Figure S7.** Intra-operative images of meniscal repair. Pre-implantation (A, C, E, G) and post-implantation (B, D, F, H) images are shown from the day of surgery for four different patients. (A, B) Patient 1 arthroscopy. (C, D) Patient 2 arthroscopy. (E, F) Patient 3 arthroscopy. (G, H) Patient 4 arthroscopy.

**Supplemental Figure S8.** MRI images for Patient 1. Sequential coronal MRI images are show for patient 1 who was unsuccessfully treated using human-MSC/collagen-scaffold therapy (re-tear requiring meniscectomy at around 15 months). A pre-operative image is shown in (A) with an arrow to indicate the torn meniscus. Post-operative images were taken at 3 months (B), 6 months (C) and 12 months (D).

**Supplemental Figure S9.** MRI images for Patient 2. Sequential coronal MRI images are show for patient 2 who was successfully treated using human-MSC/collagen-scaffold therapy. A pre-operative image is shown in (A) with an arrow to indicate the torn meniscus. Post-operative images were taken at 3 months (B), 6 months (C), 12 months (D) and 24 months (E).

**Supplemental Figure S10.** MRI images for Patient 4. Sequential coronal MRI images are show for patient 4 who was unsuccessfully treated using human-MSC/collagen-scaffold therapy (re-tear requiring meniscectomy at around 15 months). A pre-operative image is shown in (A) with an arrow to indicate the torn meniscus. Post-operative images were taken at 3 months (B) and 12 months (C).

**Supplemental Figure S11.** MRI images for Patient 5. Sequential coronal MRI images are show for patient 5 who was successfully treated using human-MSC/collagen-scaffold therapy. A pre-operative image is shown in (A) with an arrow to indicate the torn meniscus. Post-operative images were taken at 3 months (B), 6 months (C), 12 months (D) and 24 months (E).
