# Supplementary material for: Repair of Torn Avascular Meniscal Cartilage Using Undifferentiated Autologous Mesenchymal Stem Cells: From In Vitro Optimization to a First‐in‐Human Study
Source: Stem Cells Transl Med. 2016 Dec 15;6(4):1237–48. doi: 10.1002/sctm.16-0199 (PMC5442845; doi:10.1002/sctm.16-0199)
Supplement: Supplementary file 18 — Supporting Information 2 [file SCT3-6-1237-s018.docx]

**Repair of torn avascular meniscal cartilage using undifferentiated autologous mesenchymal stem cells: from *in vitro* optimisation to a first-in-human study**

**SUPPLEMENTAL METHODS**

**Implantation of ovine-MSC/collagen scaffolds and controls in sheep**

Under general anaesthesia with analgesic premedication and using aseptic technique approx 5ml bone marrow was aspirated from the iliac crest. The bone marrow was collected into a sterile syringe pre-loaded with 0.5ml of sterile 1000i.u. heparin. Cell culture and preparation of the ovine-MSC/collagen-scaffold was as described in Materials and Methods for human cells. At the time of implantation, an incision approximately 50mm in length was made in the medial para-patellar region approximately midway between the patellar tendon and the medial collateral ligament of the stifle joint centred on the joint space. The subcutaneous tissues were dissected bluntly to the joint capsule maintaining hemostasis. The joint capsule was incised to expose the medial femoral condyle, the medial meniscus and the medial proximal tibial plateau. The capsule was carefully dissected from the peripheral surface of the meniscus and the meniscus mobilised. A stifle joint distractor inserted between the intercondylar notch of the femur and tibia allowed the joint to be distracted to expose the meniscus. A number 15 scalpel blade was used to make a standardized 1 cm stab incision in the meniscus in a circumferential direction at a site approximately 25% in from the periphery to the free edge and at a point approximately mid way between the patellar tendon and medial collateral ligament. The incision was opened using a small curved hemostat. The MSC/collagen-scaffold or cell-free scaffold was pulled through the defect, the haemostat was then removed and the bandage trimmed to the femoral surface of the meniscus and to the periphery of the meniscus on the tibial aspect. In all animals, a single horizontal 5-0 Prolene suture was be placed across the defect and bandage in an “inside out” configuration and tied on the periphery of the meniscus prior to closure of the joint. At the end of the *in vivo* phase the animals were euthanased and the medial meniscus was harvested along with samples of articular cartilage, joint capsule, joint fluid and popliteal lymph node for histopathology. Meniscal repair was analysed histologically as described for the *in vitro* studies. Local tolerance of the implants was assessed by determining an “irritancy” score in the meniscal cartilage, articular cartilage, synovial fluid and lymph nodes, according to British Standard EN ISO 10993-6:2009 [24].

**Manufacture of the human autologous MSC/collagen-scaffold implant**

Bone marrow was collected from patients as described in the section below. Upon receipt at the manufacturing site each bone marrow aspirate was mixed with 20ml of freshly prepared Complete Medium consisting of Dulbecco’s Modified Eagle’s Medium – low glucose (DMEM-LG Sigma-Aldrich, Dorset, UK) containing 10% foetal bovine serum (FBS Invitrogen, Life Technologies Ltd, Paisley, UK), 4·5% Glutamax (Life Technologies, Paisley, UK) and 5ng/ml FGF-2 (Peprotech EC Ltd, London, UK) The resulting marrow-medium mix was distributed between five 175ml tissue culture flasks containing 24ml of complete media by the addition of 4ml of mix per flask. Cultures were incubated undisturbed for 4 days at 37^o^C in 5% CO_2_ and 95% air. After which time, the cells were maintained by media exchange on days 4, 7 and 10. After 13 days, the un-passaged cells were harvested using 0.25% trypsin-EDTA (Life Technologies Ltd, Paisley, UK) and their identity and purity confirmed by immunohistochemical analyses of positive and negative markers. Positive identity as mesenchymal stem cells required >80% expression of both CD105 and CD90. Confirmation of purity required <10% expression of CD34 and CD45, indicating minimal contamination with haematopoietic stem cells. The cells were then seeded onto a collagen matrix (Avitene^TM^ Ultrafoam^TM^ collagen sponge (Bard Ltd, Crawley, UK), an absorbable haemostat consisting predominantly of type I collagen derived from bovine corium) at a dose of 10^6^ cells/cm^2^ of scaffold material, and incubated at 37°C in 5% CO_2_/air for approximately 5h after which the sponge was immersed in hypothermosol (BioLife Solutions, Bothell, WA, USA).
